# A Potential Role for Felbamate in TSC- and NF1-Related Epilepsy: A Case Report and Review of the Literature

**DOI:** 10.1155/2015/960746

**Published:** 2015-10-22

**Authors:** Natanya M. Mishal, Dimitrios Arkilo, Ju Tang, John R. Crawford, Sonya G. Wang

**Affiliations:** ^1^Division of Neurology, Department of Pediatrics, Rady Children's Hospital San Diego and University of California San Diego, San Diego, CA 92123, USA; ^2^Minnesota Epilepsy Group, P.A. of United Hospital and Children's Hospitals and Clinics of Minnesota, St. Paul, MN 55102, USA; ^3^Division of Neurology, Department of Pediatrics, Tufts University School of Medicine and Floating Hospital for Children, Tufts-New England Medical Center, Boston, MA 02111, USA

## Abstract

A 15-year-old girl with maternal inheritance of neurofibromatosis type 1 (NF1) and paternal inheritance of tuberous sclerosis complex (TSC) developed intractable epilepsy at age 5. Her seizures were refractory to adequate doses of four antiepileptic medications until felbamate was initiated at age 7. She has since remained seizure-free on felbamate monotherapy. Although felbamate has multiple mechanisms of action, it is thought to have its most potent antiepileptic effects through inhibition of the *N*-methyl-D-aspartate receptor (NMDAR). Previous studies have shown that the NMDAR is altered in varying epilepsy syndromes and notably in the cortical tubers found in TSC. The aim of this paper is to examine how felbamate monotherapy was able to achieve such robust antiepileptic effects in a unique patient and possibly offer a novel therapeutic approach to patients suffering from TSC- and NF-related epilepsy.

## 1. Introduction

Tuberous sclerosis complex (TSC) and neurofibromatosis type 1 (NF1) are the two most common neurocutaneous disorders [1]. However, the occurrence of both diseases in a single individual is extremely rare and expected to occur in only one out of every 12 to 27 million people at the genotype level [2]. Both TSC and NF1 are transmitted in an autosomal dominant fashion, but there is also a high rate of new mutations among both diseases [3]. To our knowledge, the patient discussed in this paper is the only case reported in the literature with both TSC and NF1 inherited simultaneously rather than caused by sporadic mutations [3].

Of the neurocutaneous syndromes, population-based studies suggest that the occurrence of epilepsy in TSC is 78% (up to 96% in clinic-based studies) [1] and 3–6% in NF1 [4]. The patient described certainly had an elevated risk of developing epilepsy and did so at age 5. She was trialed on adequate doses of four antiepileptic medications but continued to be refractory until felbamate was initiated at age 7. She has since remained seizure-free on felbamate monotherapy.

Among other proposed mechanisms of action of felbamate, its most potent antiepileptic effects are through inhibition of the* N-*methyl-D-aspartate receptor (NMDAR). NMDARs are glutamate-gated cation channels that cause excitatory synaptic transmission and are critical for the development of the central nervous system, learning, memory, and neuroplasticity. Abnormal expression levels and altered NMDAR function have been implicated in numerous neurological disorders, including Alzheimer's disease, Parkinson's disease, neuropathic pain syndromes, psychiatric disorders, and epilepsy [5]. Focusing on epilepsy, felbamate is the only marketed anticonvulsant to date that is an NMDAR inhibitor at therapeutic concentrations that does not have serious neurobehavioral complications [6].

We report the case of a 15-year-old patient with TSC, NF1, and intractable epilepsy who only achieved seizure freedom with felbamate monotherapy and hypothesize a novel therapeutic approach to NMDAR-activated epilepsy syndromes.

## 2. Case Presentation

**Figure 1:**
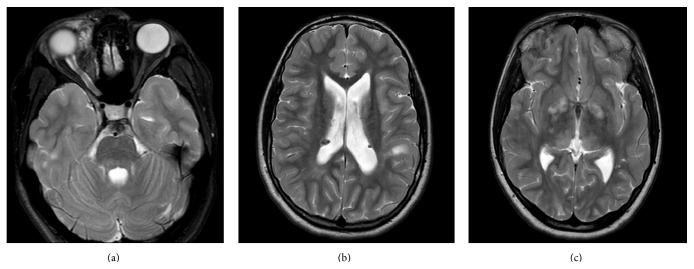
MRI images of the patient. Axial T2 images demonstrating (a) a right anteromedial orbital plexiform neurofibroma, (b) subependymal nodules and a cortical tuber, and (c) hyperintense signal in the thalamus and globus pallidus.

The patient was born at 39 weeks of gestation to a mother with NF1 and a father with TSC [3]. Physical examination at birth was significant for bilateral ear pits and one Shagreen patch on her posterior right thigh. At 1 month of age her right eye was noted to be more prominent than the left and magnetic resonance imaging (MRI) the following month confirmed proptosis without any optic nerve masses. The MRI also identified subependymal nodules and hamartomas, suggesting a diagnosis of TSC. By 4 months of age she had multiple hypomelanotic macules, confirming TSC, and more than six café-au-lait patches that, with her extensive family history of NF1 (including a first-degree relative), met criteria for a diagnosis of NF1 as well. Genetic testing was not performed, as the diagnoses were attained with satisfactory clinical criteria. The MRI was repeated at 8 months of age due to worsening proptosis and showed a developing optic nerve glioma [7]. Physical examination at age 3 displayed multiple scattered café-au-lait spots and hypopigmented macules, with new axillary and inguinal freckling and a possible early neurofibroma on her left calf   [3]. Most recent MRI of her brain at age 13 demonstrated a right orbital plexiform neurofibroma (Figure 1(a)), cortical and subcortical tubers (Figure 1(b)), subependymal nodules (Figure 1(b)), and deep white matter FLAIR and T2 hyperintensities (unidentified bright objects (UBOs) or “NF spots”) (Figure 1(c)). Cognitively, over the years, she had mild delay in meeting her developmental milestones.

**Figure 2:**
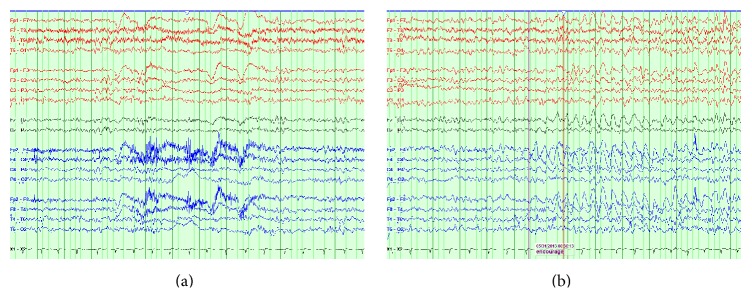
Patient's EEG displayed in a longitudinal bipolar montage showing (a) rare sharp waves in the left parietal area and (b) asymmetric slowing during hyperventilation with right-sided amplitudes greater than the left.

At 5 years of age the patient developed seizures characterized by staring and atonic falls. Her initial electroencephalogram (EEG) showed left frontal and anterior temporal spikes and sharp waves as well as intermittent focal temporal slowing. She was first treated with carbamazepine (CBZ) 200 milligrams (mg) twice daily, but over the next 5 months she continued to have breakthrough seizures as well as new seizures characterized by rhythmic head movements and eye deviation. She was changed to valproic acid (VPA) (125 mg three times daily) and clonazepam (CLN) (0.5 mg twice daily) but after 3 months required the addition of levetiracetam (LEV) as well. She continued to have breakthrough seizures so the following year at age 7, VPA and CLN were replaced by felbamate 120 mg three times daily. She remained on dual therapy LEV (up to 750 mg twice daily) and felbamate for the next 2.5 years until she was able to wean off of LEV and transition to felbamate monotherapy at 240 mg twice daily. Her most recent EEG at age 13 showed rare left parietal and bilateral frontal sharp waves (Figure 2(a)) as well as intermittent asymmetric slowing during hyperventilation (Figure 2(b)). Despite her EEG, she has since remained seizure-free on felbamate monotherapy for the last 8 years.

## 3. Discussion

### 3.1. Overview of TSC and NF1

NF1 is an autosomal dominant disorder caused by a mutation on chromosome 17q11.2, an area that codes for the tumor suppressor gene neurofibromin. Neurofibromin loss leads to upregulation of the renin-angiotensin system and hyperactivation of the mammalian target of rapamycin (mTOR) pathway [8]. This in turn causes abnormal cellular growth and proliferation. Similarly, TSC is also an autosomal dominant disorder caused by mutations in TSC1 (chromosome 9q34) or TSC2 (chromosome 16p13.3), which code for hamartin and tuberin, respectively. Together these proteins form a heterodimer that inhibits Rheb (Ras homolog expressed in brain), the GTPase that activates the mTOR pathway [8]. Deficiency in the hamartin-tuberin complex leads to hyperactive mTOR signaling and abnormal cellular division, resulting in dysgenic lesions in multiple organ systems. In the central nervous system, these include cortical tubers, radial glial bands, subependymal nodules, and subependymal giant cell tumors [9]. As described, NF1 and TSC are two examples where mutations upstream of the mTOR pathway cause dysregulation and subsequent cellular alterations that correlate clinically with epilepsy syndromes and neurodevelopmental disorders [8].

### 3.2. Epilepsy and the Approach to Treatment in TSC and NF1

Among seizures, cognitive impairment, and neurobehavioral abnormalities, epilepsy is the most common neurologic manifestation of TSC, occurring in 70–90% of patients [4]. Approximately 2/3 of patients experience seizure onset within the first year of life, typically presenting as infantile spasms or focal seizures [9, 10]. Later in the disease, focal onset seizures are more common and generalization can occur as the disease progresses due to secondary bilateral synchrony [9]. Seizures in TSC are often refractory to medications and may require resection of particular tubers. One large single-center study reported refractory epilepsy in nearly two-thirds of their cohort [9].

There are a number of proposed mechanisms by which epilepsy develops as a consequence of TSC, though cortical tubers are thought to be the largest determinant [11]. Abnormal cell types comprising the tubers likely have intrinsic epileptogenicity and cause seizures by releasing neurotransmitters or neuromodulators into adjacent brain tissue [12]. Epilepsy in TSC has also been found to be triggered by an imbalance of decreased inhibition (reduced GABA-receptor expression) and increased excitation (proliferation in the expression of glutamate receptors and subunits of the NMDAR) [12].

Classically, vigabatrin, an irreversible inhibitor of GABA-transaminase (the enzyme responsible for catabolism of GABA), has been used as the first line for TSC-associated infantile spasms. After a period of remission from spasms, focal and then generalized seizures usually begin to appear and can become quite severe and unremitting, requiring combinations of multiple antiepileptic medications [12]. Other than vigabatrin, which has distinctly been documented to be effective in TSC-associated infantile spasms, there is no clear evidence for the superiority of one antiepileptic drug over another. For those patients refractory to medical therapy, alternative options include ketogenic diet or epilepsy surgery when epileptogenic regions can be localized to singular tubers.

Neurological manifestations of NF1 mainly include intracranial tumors such as optic gliomas, focal areas of T2-hyperintensity (UBOs), and intellectual and learning disabilities [13]. The prevalence of epilepsy in NF1 is relatively low compared to TSC and other neurocutaneous disorders, occurring in only 3–6% of patients [4]. Interestingly, in the context of our patient, one study concluded that individuals with seizures were more likely to have inherited NF1 from their mother [14]. The cause of seizures in NF1 has not been clearly demonstrated and the relationship between T2-hyperintensities and seizures is controversial. One study found that there is an increased risk of epilepsy related to intracranial tumors and cytoarchitectural abnormalities such as cortical malformations and mesial temporal sclerosis [13]. Conversely, another study did not find any relationship between the presence of subcortical focal brain lesions and seizure type, response to treatment, or evolution of epilepsy [13]. Generally, when seizures do occur in NF1, they are thought to be fairly easy to control with 60–70% of individuals requiring one or even no antiepileptic medications [4]. Medication selection is based on seizure type and thus there is no algorithm specific to NF1.

### 3.3. Crossroads between Epilepsy and the NMDAR

The NMDAR is fundamental to excitatory neurotransmission and is critical for normal CNS function. The receptor is a glutamate-gated cationic channel composed of four polypeptide subunits around a central pore, including at least two obligatory NR1 subunits with variable expression of two of the four types of NR2 subunits (NR2A-D) [6]. The variability of the NR2 subunit confers unique pharmacological and biophysical properties upon the NMDARs that they form. The channel is activated when glutamate released from the presynaptic terminal diffuses across the synaptic cleft and binds to its site on the NR2 subunit [5]. Glycine acts as a coagonist for glutamate; thus the glycine binding site on the NR1 subunit must be occupied before glutamate can bind to its site on NR2. Once the receptor is activated, Ca^2+^ and Na^+^ are conducted across the channel, resulting in excitatory postsynaptic potentials. Mg^2+^ plays an important role in blocking the channel and preventing ion permeation [5].

Aberrant NMDAR function is implicated in a wide range of CNS disorders, including acute and chronic pain syndromes, stroke, head trauma, dementias, and epilepsies [15]. In cerebral ischemia, dying neurons release glutamate which overactivate neurons in the penumbra, whereas compromised neurons are more susceptible to excitotoxic damage in neurodegenerative disorders such as Alzheimer's and Parkinson's diseases [16]. In epilepsy (and in neuropathic pain syndromes), there is overactivity of excitatory pathways. When the NMDA channel is overstimulated and Ca^2+^ and Na^+^ are conducted in excess, there is resultant neuronal excitotoxicity and subsequent seizure discharges [6].

Multiple investigators have shown that the NMDAR is altered in patients with epilepsy. One study demonstrated that increased phosphorylation of the NR2B receptor in vivo resulted in epileptic discharges [17]. Another reported that hippocampal mRNA levels of the NR2 subunit were increased in patients with hippocampal sclerosis [18]. It was concluded by other studies that NMDARs are increased in dentate granule cells in patients with temporal lobe epilepsy [19]. More recent studies have shown an upregulation of the composition of the NR2B subunit in addition to altered sensitivity to Mg^2+^ blockade in children with cortical dysplasias and adults with temporal lobe issues [20, 21]. Furthermore, many studies utilize low Mg^2+^ in vitro models of epilepsy whereby decreased NMDAR blockade induces epileptiform activity [22, 23]. In status epilepticus, prolonged seizures have been found to cause upregulation of NMDARs [24]; as glutamate continues to activate the receptors there is an influx of more and more Ca^2+^ and Na^+^ which further potentiates epileptogenicity. Additionally, anti-NMDA receptor antibody encephalitis is considered a relatively new entity in which antibodies are formed against the NR1 subunit of the NMDAR, subsequently causing a decrease in cell surface NMDAR expression and impaired glutamate regulation [25]. This results in a severe encephalitis marked by psychiatric symptoms, memory issues, seizures, dyskinesias, and autonomic dysfunction [26].

In reference to TSC, one study by White et al. [27] examined alterations in receptor expression in cortical tubers and found that there was an increase in NR2B and 2D subunit mRNAs. This in turn caused an increase in functional NR2B-containing subunits on the NMDAR and thus an increase in excitatory transmission mechanisms. Using TSC as a model for malformations of cortical development such as focal cortical dysplasias (FCD), another study found that NMDARs of pyramidal neurons in FCD showed decreased sensitivity to Mg^2+^ inhibition, thus promoting neuronal excitability [28]. These findings are thought to contribute to the epileptogenesis of cortical tubers and suggest that NMDAR subtype-selective medications may be of particular value in treating seizures in TSC.

There are no studies to date that specifically examine alterations in the NMDAR in NF1. Neurofibromin has, however, been documented to interact with a number of molecules including NMDARs whereby NMDAR activation precedes neurofibromin inactivation of Ras, subsequently allowing for synaptic plasticity, structural plasticity (specifically in dendritic spines), and new spine formation [29, 30]. Loss of neurofibromin as in NF1 causes sustained Ras activation with impaired plasticity and loss of spines, which has been thought to at least partially explain the learning disabilities associated with the disease [30].

### 3.4. NMDA Receptor Antagonists

NMDAR antagonists were first described to possess anticonvulsant activity in 1982 [31]. This has since been confirmed in multiple studies using experimental seizure models. The use of the NMDAR antagonist ketamine for refractory status epilepticus has been thoroughly examined in the literature, both in animal and human models. Among the studies, 50–60% of patients had cessation of their refractory status epilepticus with ketamine administration [32].

However, in clinical trials, NMDAR antagonists have demonstrated serious and unacceptable neurobehavioral side effects at therapeutic doses [33], including hallucinations, catatonia, ataxia, nightmares, and memory deficits [5]. To date, the only approved anticonvulsant that inhibits the NMDA channel at therapeutic concentrations is felbamate [34]. Although serious side effects such as aplastic anemia and hepatotoxicity have limited its use, the anticonvulsant efficacy of felbamate makes it too important of a drug to discard.

### 3.5. Felbamate Indications and Safety Profile

Felbamate (FBM, 2-phenyl-1,3-propanediol dicarbamate) was synthesized in 1955 but was first approved in 1993 as a novel antiepileptic to be used as monotherapy and as an adjunctive therapy to treat partial seizures with and without generalization in adults and as an adjunctive therapy for partial and generalized seizures associated with Lennox-Gastaut syndrome [35, 36].  Felbamate has been shown in animal models to increase the after-discharge threshold of seizures, reduce seizure severity, and shorten seizure duration [35]. In humans, there have been double-blinded, randomized, placebo-controlled monotherapy clinical trials that have proven effective in reducing seizure frequency in severe refractory epilepsy [35].

Despite its favorable efficacy, there has been restricted approval of felbamate to only adjunctive therapy in patients with Lennox-Gastaut syndrome because of postmarketing experience suggesting felbamate-related idiosyncratic aplastic anemia and hepatotoxicity [37]. In the first year of availability, more than 110,000 patients received felbamate. There were 23 confirmed cases of aplastic anemia, but only 3 were definitely related to felbamate. No cases were reported in patients less than 13 years of age. From this data, the estimated risk of aplastic anemia was determined to be 127 cases per million patients who take felbamate. Additionally, five deaths from felbamate-associated liver failure were reported, placing the estimated incidence at 1 in 26,000–34,000. This is compared to 1 in 10,000–49,000 associated with valproic acid, an antiepileptic drug more commonly known to cause hepatotoxicity [36]. More frequently reported side effects of felbamate, however, include anorexia, weight loss, nausea, insomnia, dizziness, and headache [38].

### 3.6. Felbamate Mechanism of Action

Although the exact mechanism of action is unclear, the major antiepileptic effect of felbamate is at least partly due to inhibition of the NMDAR, which decreases excitatory neurotransmission [35]. Felbamate has also been shown to inhibit voltage-gated sodium channels, block voltage-sensitive calcium channels, and potentiate GABAergic activity [36]. However the effect on GABA function is relatively minor, as shown through studies demonstrating decreased effectiveness of felbamate as an anticonvulsant for seizures induced by GABA-blockade [39]. This may also help explain why felbamate does not have the severe sedative or cognitive side effects possessed by other anticonvulsants.

At the NMDAR, therapeutic concentrations of felbamate have an inhibitory effect on NMDA currents [40]. However, the binding affinity and action of felbamate vary according to drug concentration, the gating state of the channel, and the ambient concentrations of NMDA and glycine [34]. When NMDA concentrations are low, felbamate acts as a partial allosteric agonist, enhancing NMDA binding, channel opening, and excitatory conduction. However, felbamate has a much higher affinity for open NMDA channels, whereby conformational changes of the NR1-NR2 complex presumably make the drug binding site more accessible and allow felbamate to bind and block pore currents [34].

The exact site of felbamate action on the NMDAR is still unknown, but a number of studies have offered hypotheses, including selective blockade of specific subunits [31], modification of the glycine binding site [41], or acting through an open channel block similar to how Mg^2+^ inhibits the channel [42]. Though felbamate is able to inhibit receptors composed of varying subunits, its most potent antagonistic effect occur with NR2B-containing heteromers [31, 43]. This selectively for the NR2B subunit is remarkable in the context of TSC-related epilepsy, where TSC studies have shown an increase in NR2B mRNA and thus NR2B subunit production [27]. This also may help explain the efficacy of felbamate in childhood epilepsies (such as Lennox-Gastaut syndrome), as the developing brain has an abundance of diffusely distributed NR2B subunits in contrast to adults where NR2B subunits become largely restricted to the forebrain and hippocampus [31].

## 4. Conclusions

### 4.1. Could Felbamate Be a Novel Therapeutic Option for Patients with TSC- and NF1-Related Epilepsy?

As described, the majority of patients with TSC develop epilepsy whereas the prevalence of epilepsy in NF1 is only slightly above the 1-2% value reported for the general population. The patient described in this paper carries a diagnosis of both TSC and NF1. Presumably it was the TSC that primarily generated her refractory epilepsy, but the NF1 likely potentiated her risk. She was tried on a number of antiepileptic medications, both as monotherapy and in combination, but failed to gain seizure control until felbamate was initiated.

There are a number of proposed mechanisms by which TSC causes epilepsy, of which upregulation of excitatory mechanisms such as NMDAR expression is an interesting theory in the context of this patient. Multiple studies have demonstrated that the NMDAR is altered in epilepsy syndromes including hippocampal sclerosis, cortical dysplasia, temporal lobe epilepsy, refractory status epilepticus, and anti-NMDA receptor antibody encephalitis.

Of particular interest is the study by White et al. [27] that found an increase in NR2B NMDAR subunits in cortical tubers in TSC. With the knowledge that the major antiepileptic effect of felbamate is due to its inhibition of the NMDAR, specifically at the NR1 and NR2B subunits, we can speculate that this may have been the reason for the robust antiseizure response experienced by our patient to felbamate. Similarly, in anti-NMDA receptor encephalitis, it has been found that there is strong expression of the NR2B (and NR2A) subunits in teratomas associated with the disease [44]. There is one patient with anti-NMDA receptor encephalitis reported in the literature that had multidrug-resistant nonconvulsive status epilepticus that resolved only after the addition of felbamate [45].

The patient described had been tried on carbamazepine (CBZ), valproic acid (VPA), clonazepam (CLN), and levetiracetam (LEV) but did not achieve seizure freedom until felbamate was added. Though felbamate functions through similar mechanisms of action as these other drugs including inhibition of voltage-gated sodium channels (CBZ, VPA), increasing GABA (CBZ, VPA, LEV, and CLN), and blocking calcium channels (VPA), the only mechanism of action exclusive to felbamate is its inhibition of the NMDAR [46]. Perhaps this is why felbamate worked and the others did not.

### 4.2. The Many Faces of the NMDAR

In both TSC and NF1, chromosomal mutations cause abnormal formation of mTOR inhibitory proteins and hyperactivation of the pathway. Abnormal cell growth and proliferation due to mTOR hyperactivation promote seizures by indirectly affecting the excitability of circuits through alterations in neurotransmitter and ion channels and neuronal and synaptic organization [47, 48]. Both in vitro and human models have suggested that this hyperactivity is at least partially responsible for the developmental abnormalities and epilepsy seen in these conditions.

The structure and function of the NMDAR were explained above in detail; however one of the faces of the NMDAR is as an important regulator of the mTOR pathway. The receptor works upstream of mTOR to downregulate the pathway and, thus, protein synthesis responsible for cellular growth and function [49]. There have been a number of studies showing that NMDAR antagonists rapidly activate the mTOR pathway. This idea has received particularly significant attention with the use of low dose ketamine (a nonselective NMDAR antagonist) to cause rapid antidepressant effects by activating the mTOR pathway specifically in the prefrontal cortex of rats [50]. However, it is interesting that ketamine has potent antiepileptic qualities as well [51]. Raising further questions is the fact that antibodies in anti-NMDA receptor encephalitis are known to cross-link and internalize the receptor, causing subsequent receptor hypofunction. This theory of hypofunction supports the psychotic features but fails to explain why many patients have seizures in the early stages of the disease [52]. In particular, it is counterintuitive that at least one patient reported in the literature achieved seizure freedom with use of felbamate, which would have caused further NMDAR antagonism [45].

These unexpected outcomes raise questions as to how NMDA inhibitors such as felbamate can have such potent antiepileptic effects if NMDA inhibition is thought to augment the mTOR pathway and thus promote epileptogenesis. As discussed above, felbamate has a number of mechanisms of action [36] and, at least at the NMDAR, the exact site of action is still unknown. Furthermore, these mechanistically unexpected effects may be attributable to the dose dependence of felbamate, whereby the binding affinity varies according to drug concentration, channel state, and ambient NMDA concentration [34]. The efficacy of felbamate in our case is more likely due to the intrinsic epileptogenicity within the intracranial dysgenic lesions [12] and alteration of NMDARs within those lesions [27–30] rather than targeting the receptors upstream of the mTOR pathway. Undoubtedly, more information is needed to explain the role of the NMDAR in the mTOR pathway and how dysregulation can promote epileptogenesis.

### 4.3. Future Directions

Although our findings are based on the experience of only one patient, the robust response and overlap between the mechanical underpinnings of the disease and the drug suggest that perhaps felbamate should be considered in the treatment of TSC-related epilepsy. Vigabatrin has proven to be effective in infantile spasms in TSC by upregulating GABA. Perhaps the addition of felbamate can help treat the succeeding refractory seizures in TSC by targeting the NMDAR as well. Though less is known about the cause of seizures and the role of the NMDAR in NF1, it has been documented that neurofibromin and the NMDAR do in fact interact. Interestingly, there is one case report on the use of ketamine, an NMDA antagonist, for a patient with severe chronic pain secondary to NF1 that was refractory to multiple therapies but responded quite dramatically to ketamine [53]. Perhaps this suggests an upregulation of NMDARs in NF1 as well and potential for response to NMDA-targeted drugs.

Clearly large clinical study would be ideal to address our hypothesis, but further publications of clinical reports or case studies can begin to add depth to our proposal. As a final point, more research is also needed about the potential role of felbamate or other NMDAR antagonists in the varying epilepsy syndromes described above that have also been documented to have alterations in the NMDAR.
